# No Childhood Advantage in the Acquisition of Skill in Using an Artificial Language Rule

**DOI:** 10.1371/journal.pone.0013648

**Published:** 2010-10-27

**Authors:** Sara Ferman, Avi Karni

**Affiliations:** 1 Department of Communication Disorders, Sackler Faculty of Medicine, Tel Aviv University, Tel Aviv, Israel; 2 Department of Human Biology, Faculties of Science and Education, Edmond J. Safra Brain Research Center for Learning and Learning Disabilities, University of Haifa, Haifa, Israel; University of Groningen, Netherlands

## Abstract

A leading notion is that language skill acquisition declines between childhood and adulthood. While several lines of evidence indicate that declarative (“what”, explicit) memory undergoes maturation, it is commonly assumed that procedural (“how-to”, implicit) memory, in children, is well established. The language superiority of children has been ascribed to the childhood reliance on implicit learning. Here we show that when 8-year-olds, 12-year-olds and young adults were provided with an equivalent multi-session training experience in producing and judging an artificial morphological rule (AMR), adults were superior to children of both age groups and the 8-year-olds were the poorest learners in all task parameters including in those that were clearly implicit. The AMR consisted of phonological transformations of verbs expressing a semantic distinction: whether the preceding noun was animate or inanimate. No explicit instruction of the AMR was provided. The 8-year-olds, unlike most adults and 12-year-olds, failed to explicitly uncover the semantic aspect of the AMR and subsequently to generalize it accurately to novel items. However, all participants learned to apply the AMR to repeated items and to generalize its phonological patterns to novel items, attaining accurate and fluent production, and exhibiting key characteristics of procedural memory. Nevertheless, adults showed a clear advantage in learning implicit task aspects, and in their long-term retention. Thus, our findings support the notion of age-dependent maturation in the establishment of declarative but also of procedural memory in a complex language task. In line with recent reports of no childhood advantage in non-linguistic skill learning, we propose that under some learning conditions adults can effectively express their language skill acquisition potential. Altogether, the maturational effects in the acquisition of an implicit AMR do not support a simple notion of a language skill learning advantage in children.

## Introduction

A widely held notion posits that the ability to learn a language declines between childhood and young adulthood [1]–[6]. This notion of childhood superiority in language learning has been related to ‘critical’ or ‘sensitive’ early-life periods wherein neuronal properties are particularly susceptible to shaping by experience, i.e., windows of opportunity for skill, including language, acquisition; subsequently, with maturity, there is reduced ability for neural plasticity [7]–[9]. Another notion, related to the neurobiological tenet of two long-term memory systems, suggests that childhood superiority in the acquisition and retention of language skills reflects a shift from reliance on procedural memory in childhood to greater dependency on declarative memory in adulthood [5]–[6]. According to this view, language learning is less efficient in adults because different memory mechanisms dominate.

A leading tenet is that two independent neural systems subserve long-term memory: the declarative and procedural memory systems [10]–[11]. Declarative memory has been implicated in the learning and subsequent use of knowledge about events and facts (‘what’). This type of memory can be established following even a single exposure and explicitly recollected, but may be rapidly degrade. The procedural memory system has been implicated in the learning and retention of skills (‘how to’) and habits, and its establishment necessitates a critical amount of repetition (practice) and time [12]–[14]. The establishment of procedural memory is sometimes conceptualized as implicit learning, i.e., the acquisition of complex structured knowledge independently, to a large degree, of awareness of both the processes and products of acquisition [15]–[16]. Explicit learning, in this view, may be more intentional, conscious and reportable [10]–[11]. Verbal reporting, therefore, is considered a relatively sensitive measure for demarcating implicit and explicit memory in humans [15]–[16].

There is evidence suggesting that whereas procedural memory matures early on in childhood, declarative memory develops later during childhood and matures in adolescence [17]–[19]. It has been proposed that language, especially grammar and pronunciation, is highly appropriate for procedural (implicit) learning [5]–[6].

Much of the evidence for the notion that ‘earlier is better’ in language learning comes from studies showing a negative correlation between the learner's age at acquisition and the proficiency attained in a first language [20]–[21], a second language [1]–[6], [22]–[23] and sign language [24]–[25]. In second language acquisition, childhood superiority was specifically found for grammar [1]–[3], [26] and pronunciation [23], [27]–[28]. However, others have shown that some late second language learners can attain language proficiency that is equal or superior to that of early learners [4], [28]–[34] including in aspects such as grammar [29]–[32] and pronunciation [28]–[29], [34]. It has been proposed that the ‘early is better’ notion, in terms of speech and language achievements, may reflect environmental and cognitive factors such as the amount and duration of practice [23], [35], the level of education, quantity and quality of input [35]–[37] and interference [38]–[40] rather than sensitive period constraints. Several neuro-imaging studies also suggest that the identification of different brain activation foci for early and late second language acquisition [41]–[42] may reflect factors such as the amount and duration of experience or the nature of the language experience rather than a childhood window of opportunity [43]–[46].

Only a few studies have investigated differences in language skill acquisition as a function of maturation, under equivalent language learning conditions. A seminal study by Asher & Price [47] in which 8-, 10- and 14-year-old English-speaking children and young adults received an equivalent, albeit relatively short, learning experience in learning to comprehend Russian statements, reported a clear age effect with the adults outperforming the children. For non-linguistic skills, the notion of a highly effective skill learning capacity in adults as well as a robust capacity for experience-dependent neural plasticity is well supported [12]–[14], [ 48]–[49]. A recent study has demonstrated no childhood superiority in either learning, consolidation, or retention of a motor skill [50], although adults manifested more susceptibility to interference, i.e., they were more selective, rather than less effective, in establishing long-term memory [50]. Brain imaging studies have shown significant experience-dependent brain activation changes for language related skills in adults [51].

The aim of the current study was to test the hypothesis of childhood advantage in learning a new linguistic skill when participants of different age groups – 8- and 12-year-olds and young adults – are provided with an equivalent multi-session learning experience, controlling for factors such as the novelty of the to-be-learned task and the amount and duration of exposure and practice. Our findings support the notion of a maturational effect between childhood and adulthood in the establishment of declarative but also procedural memory when learning a complex artificial language task.

## Materials and Methods

### Ethics statement

The study was approved by the University of Haifa Ethics committee. A written informed consent was obtained from participants or in the case of children from both parents.

### Participants

Twenty-four healthy participants, eight from each age group (four males and four females): 8-year-olds (mean 8.02 years), 12-year-olds (mean 12.03 years) and young adults (mean 21.05 years) participated in the study. All participants were healthy native Hebrew speakers from middle-class backgrounds, with no reported history of speech, language, learning or hearing difficulties.

### Materials

The artificial morphological rule (AMR) was designed to be analogous to the morphological rules of Hebrew grammar. The AMR required a specific differential phonological marking for Hebrew verbs depending on whether the preceding noun (the subject) was animate or inanimate. This semantic distinction is not expressed in Hebrew; nevertheless, in some natural languages (including English) an animate inanimate distinction is made in some manner. Thus, in the AMR, the suffix/***ev***/was to be added to verbs used with animate nouns and the suffix/***ar***/was to be added to verbs used with inanimate nouns. Also, in accordance with Hebrew phonological rules, all transformed verbs underwent omission of the vowel that preceded the added suffix and hence the stress shifted to the added suffix. The AMR was applied to grammatical Hebrew noun-verb phrases (items) so that the meaning of the phrases could be easily understood by 8-year-olds. To test whether 8-year-olds can clearly distinguish between living and non-living objects, forty 8-year-olds, who did not take part in the learning study, were asked to indicate whether each item on a list of 20 common nouns was animate or inanimate. The group average score was 95%.

### Stimuli

Four types of item lists were used: (1) modeling lists – each included 16 noun-verb pairs that were well constructed according to the AMR. (2) Repeated-item lists – each included the same 16 items as the modeling lists, but with each item repeated twice in a given list (32 items). These lists were used to test for the ability to learn specific items. (3) Pre-test lists – each included the 16 items from the repeated-item list. The order of the items in all lists was randomized in each presentation. (4) New-item lists – each included 16 new noun-verb pairs, with each item presented only once during the whole study. These lists were used to test for the ability to generalize the AMR to previously un-encountered items. In each list of every type, there were equal numbers of animate and inanimate nouns. Three types of stimulus sets were used: (a) Well-constructed noun-verb pairs using the artificial rule; these were used in the model list and as the correct options in the judgment task (see below). (b) Noun-verb pairs that were well constructed phonologically but illegally constructed semantically that were used as the incorrect options in the judgment task. (c) Noun-verb pairs in standard Hebrew; these were used in the production task.

### Procedure

**Figure 1:**
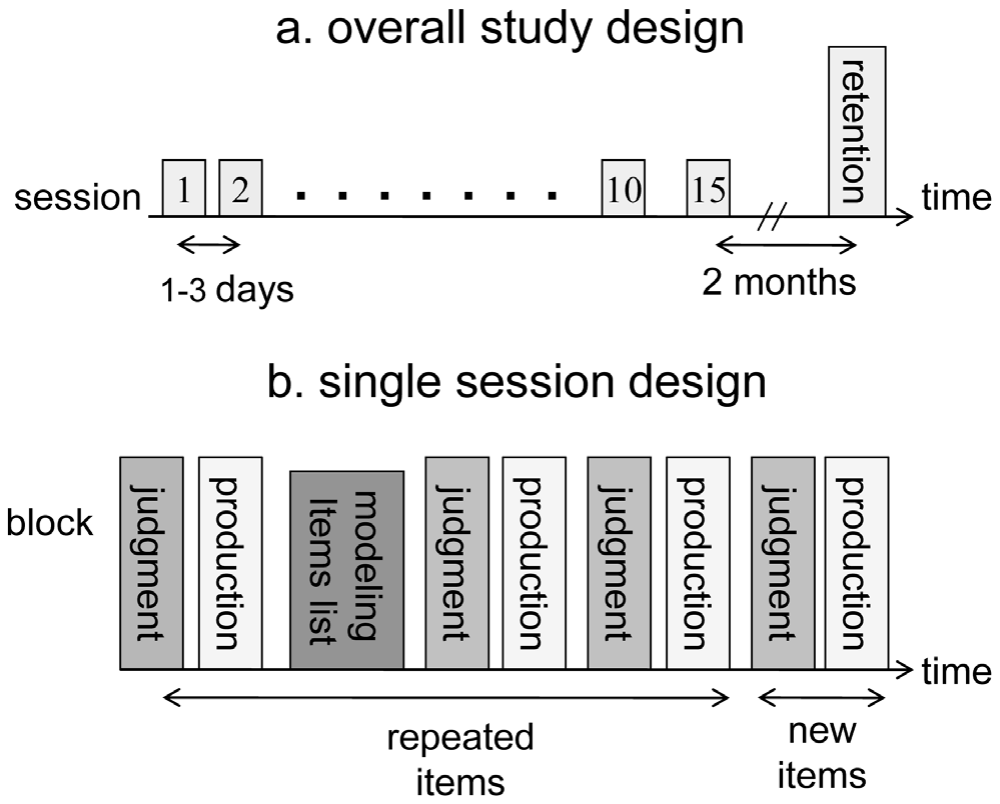
The study design. (a) The overall study design – included 10 consecutive learning sessions, 1–3 days apart, and a retention test session after an interval of two months. (b) A single session design: Two item list types (repeated and new) and two tasks (judgment and production). Each block (list) of items is represented by a column with the corresponding task.

Each participant was individually trained in 10 consecutive daily training sessions (1–3 days apart) and re-tested for retention after an interval of two months (Fig. 1a). The 8-year-olds and 4/8 of the 12-year-olds who were less than 80% correct in the production task in the 10^th^ session received five additional training sessions. At the beginning of the first session, in a pre-recorded introduction, each participant was told that he/she was *going to learn a new language, similar to Hebrew* and then instructed to listen to the modeling list in order to learn the ‘new language’. Instructions on how to respond in each of the two tasks then followed. Training occurred through exposure to and use of the AMR in the performance of two tasks: (1) a judgment task wherein the participants were instructed to make a forced-choice (correct – incorrect) response by pressing one of two buttons: (2) a production task wherein the participants were instructed to produce (voice) the transformed verb in accordance with the AMR. In the judgment task the participants heard: *In the new language, is it correct to say* … followed by a noun-verb pair, either well-constructed or not. The incorrect option was always a phonologically correct production that was semantically incorrect. In the production task the participants heard: *In the new language, how should one say* … followed by a noun and was required to produce a verbal response: the transformed verb in accordance with the AMR. In both tasks the participants were instructed to respond as quickly as possible. A pre-recorded auditory feedback signal consisting of the word *error* was automatically provided following each incorrect answer in both tasks. There was no explicit instruction on the nature of the AMR at any time during the training.

Each session included: listening to a modeling list, four blocks of repeated-item lists (two for each task), and two blocks of new-item lists (one for each task). Starting from the second session, two pre-test lists (one for each task) were administered at the beginning of each session (before the modeling list) to test between-session gains (Fig. 1b). Rest intervals of 2-3 minutes were given between blocks. Each session lasted about one hour and included 176 repeated items (80 in each task and 16 in the model list), and 32 new items (16 in each task). The first session included the same number of new items but only 144 repeated items, because there were no pre-test lists. The order of the tasks (judgment or production first) and the order of type of items (new or repeated) within a given task were pseudo-randomized for each session. There were three versions of the order of tasks and type of items (repeated or new), with each participant trained in a given version throughout.

### Apparatus and Measurements

The SuperLab software package (Cedrus Corporation, www.cedrus.com) was used to run the experimental trials and to time and log responses for each trial using a PC laptop. All stimuli and instructions were recorded by a professional radio announcer using the Goldwave software (www.goldwave.com). The length (stimulus duration) of all items was equalized. Stimuli were presented through a headphone set. Speech production answers were recorded by a microphone placed on an adjustable stand in front of the participant and voice onset measured using a Mel Response Box (Psychology Software Tools, Inc). Accuracy (correct/incorrect) and speed (Reaction Time [RT] in ms) for both manual (judgment task) and voiced (production task) responses were logged for every single response. RT was measured as the interval between the end of the verbal test stimulus and the onset of button press for the judgment task and the onset of speech production for the production task. Only very extreme RT scores in a block (twice the mean performance) were removed; these accounted for about 0.3% of the data. The average accuracy (% correct) and speed for each block (list) and each session were calculated for each task separately. Within-session (early) gains were calculated as the difference between the first (pre-test) block and the final block of the session. Between-session gains were calculated as the difference between the final block of a given session and the first block of the following session (delayed gains).

A verbal report concerning the participants' insights on the required transformation was elicited and used to assess the explicit knowledge of the AMR. At the end of each learning session, the participants heard: *You are doing very well. How did you arrive at your answer?* The verbal reports were recorded using an audio tape-recording system and written down on a pre-prepared form for off-line analysis. Although the introduction of the explicit verbal reports might have affected the learning process in calling for self- awareness and encouraging introspection on the nature of the task, it is an accepted procedure in investigating the involvement of implicit versus explicit knowledge [41].

### Statistical Analysis

Repeated measures ANOVAs were used to assess learning and retention with both session and block as within-subject factors and age group (8-year-olds, 12-year-olds and adults) as a between-subjects factor. This analysis was performed for each measure (accuracy; speed), in each task (judgment; production), for each type of items (repeated; new) separately. A similar analysis was performed for phonological accuracy in the production of new items. Pair-wise comparisons between levels of main effects were performed and p values were adjusted for multiple comparisons, using Hochberg's GT2 method.

## Results

**Figure 2:**
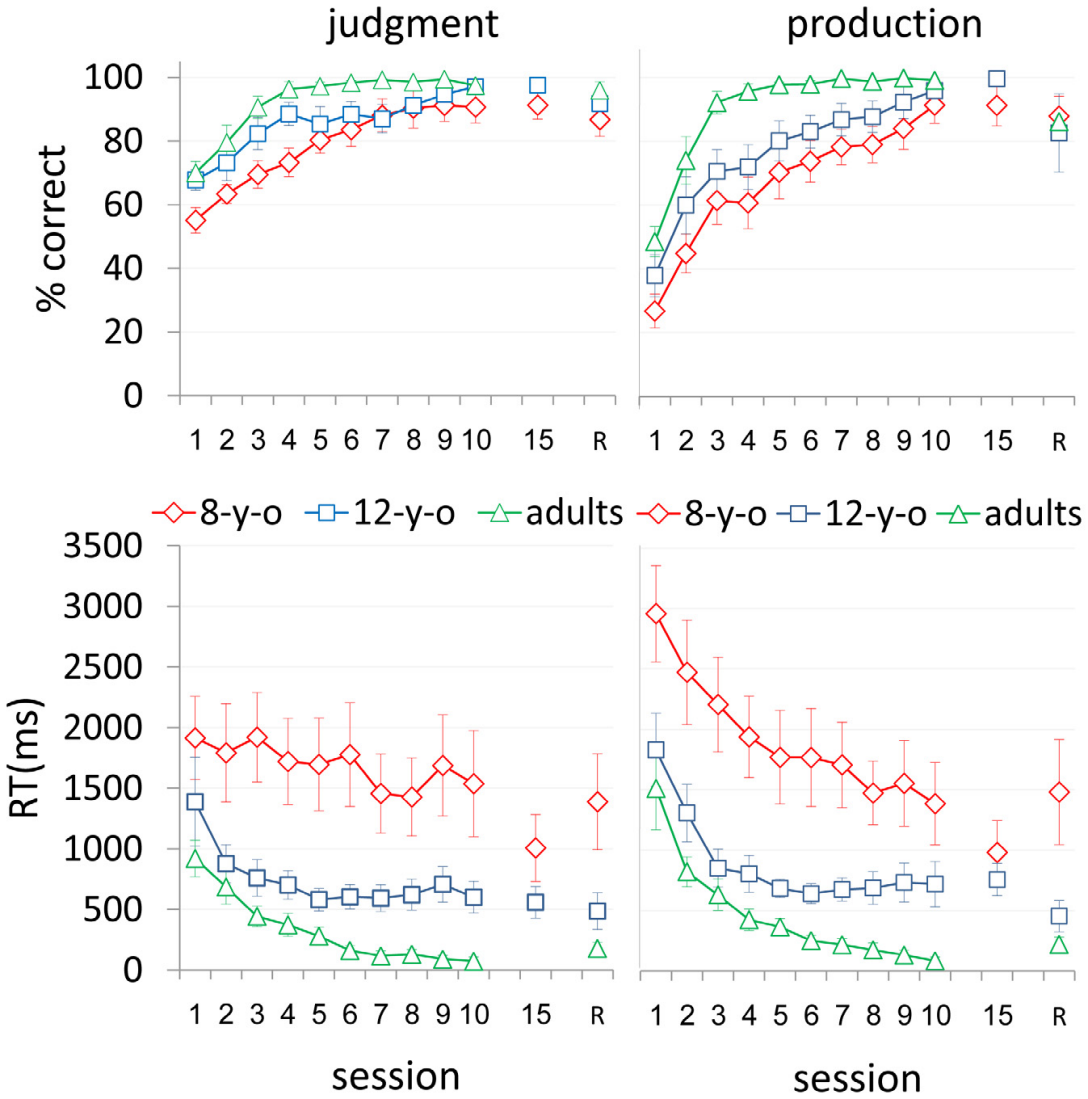
Performance on the repeated items in the 3 age-groups. Group average performance in 10 consecutive practice sessions, the 15^th^ session, and a retention session (R) in eight-year-olds (8-y-o), 12-year-olds (12-y-o) and young adults (adults). Judgment (left panels) and speech production (right panels) tasks. Accuracy (% correct) - top panels; speed [RT (ms)] - bottom panels. Error bars  =  standard errors.

**Figure 3:**
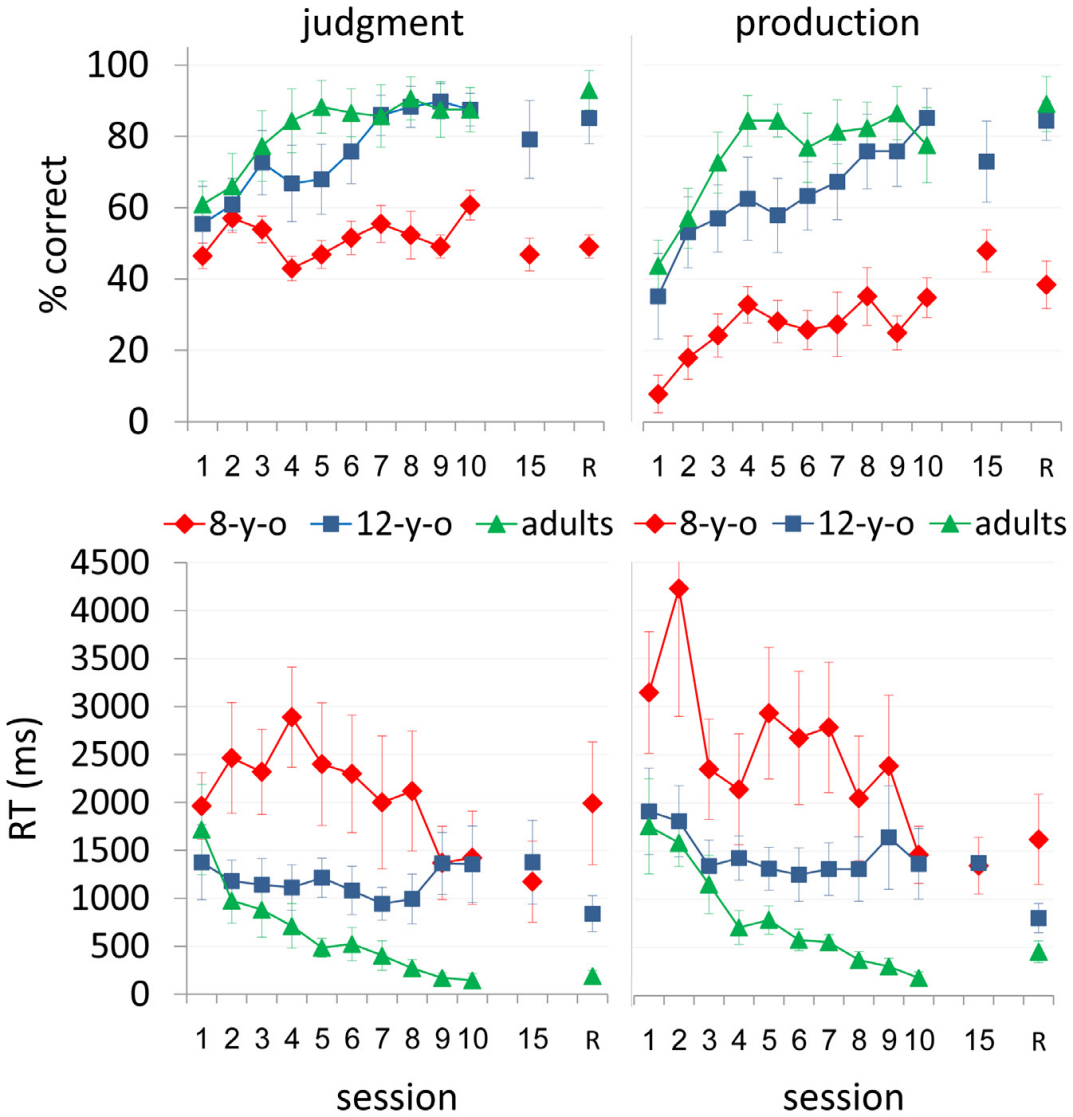
Performance on the new items in the 3 age-groups. Group average performance in 10 consecutive practice sessions, the 15^th^ session, and a retention session (R) in eight-year-olds (8-y-o), 12-year-olds (12-y-o) and young adults (adults). Judgment (left panels) and speech production (right panels) tasks. Accuracy (% correct) - top panels; speed [RT (ms)] - bottom panels. Error bars  =  standard errors.

All three age groups tested showed robust incremental performance gains for the repeated (Fig. 2) as well as the new (Fig. 3) artificial morphological rule (AMR) transformed items. The adults' performance was superior to that of the children of both age groups. The 8-year-olds were the poorest performers (in terms of speed as well as accuracy) in the initial session and attained the lowest gains in producing and in judging both repeated and new items throughout the training period. The adult advantage was clear even after the 8-year-olds were given five additional practice sessions.

### Repeated Items

For repeated items (Fig. 2), the analyses showed a main effect for session [accuracy (*F*_9,179_  = 16.43, *P*<.0001; *F*_9,180_  = 27.93, *P*<.0001); speed (*F*_9,180_  = 2.18, *P* = .0255; *F*_9,176_  = 18.09, *P*<.0001), judgment and production tasks, respectively] and for block [accuracy (*F*_2,42_  = 13.46, *P*<.0001; *F*_2,42_  = 24.67, *P*<.0001); speed (*F*_2,42_  = 9.53, *P*<.0004; *F*_2,42_  = 3.83, *P* = .0295), judgment and production tasks, respectively]. Moreover, there was a significant age-group effect for session [accuracy (*F*_2,21_  = 12.33, *P* = .0003; *F*_2,21_  = 13.5, *P* = .0002); speed: (*F*_2,21_  = 22.43, *P*<.0001; *F*_2,21_  = 28.81, *P*<.0001), judgment and production tasks, respectively], with adults outperforming both the 12-year-olds [accuracy: (*t*_21_  = −2.54, *P* = .0543); *t*_21_  = −2.29, *P* = .0923); speed: (*t*_21_  = 4.7, *P* = .0004); *t*_21_  = 5.35, *P*<.0001), judgment and production tasks, respectively] and the 8-year-olds [accuracy: (*t*_21_ = −4.97, *P* = .0002); *t*_21_ = −5.19, *P*<.0001); speed: (*t*_21_  = −4.97, *P* = .0002); *t*_21_ = 7.32, *P*<.0001), judgment and production tasks, respectively]. The 12-year-olds outperformed the 8-year-olds on accuracy but not in speed [accuracy: (*t*_21_  = −2.48, *P* = .0623); *t*_21_  = −2.95, *P* = .0226)]; [speed: (*t*_21_  = 1.85, *P* = .2097; *t*_21_  = 2.09, *P* = .1361)], judgment and production tasks, respectively]. There were no significant interactions between age group and session. When the data were split by age group, there was a main effect for session in terms of accuracy in the 8-year-olds (*F*_9,179_  = 6.62, *P*<.0001; *F*_9,180_  = 13.46, *P*<.0001, judgment and production tasks, respectively), 12-year-olds (*F*_9,179_  = 6.00, *P*<.0001; *F*_9,180_  = 8.44, *P*<.0001, judgment and production tasks, respectively) and adults (*F*_9,179_  = 5.33, *P*<.0001; *F*_9,180_  = 9.05, *P*<.0001, judgment and production tasks, respectively). There was also a main effect for session in terms of speed but only in the production task in the 8-year-olds (*F*_9,176_  = 3.87, *P* = .0002), 12-year-olds (*F*_9,176_  = 2.23, *P*<.0224) and adults (*F*_9,176_  = 2.79, *P*<.0044).

The five additional practice sessions that were given to the 8-year-olds and the four lowest achieving 12-year-olds resulted on average in improvement of speed of response (Fig. 2). A Wilcoxon Signed Rank Test however, showed that the performance in session 10 was not significantly different from that of the 15^th^ session in terms of both speed and accuracy in the two tasks. A Kruskal-Wallis non-parametric analysis of variance showed a significant difference between the three age groups in the final practice session (i.e., session 15 for 8-year-olds and 4/8 of the 12-year-olds and session 10 in adults and 4/8 of the 12-year-olds) in terms of speed in both tasks, but not in terms of accuracy. Thus, eventually the children became accurate, although less than adults, but remained slower compared to adults in both tasks (Fig. 2).

In all three age groups tested, average performance in the final practice session did not differ significantly from performance in the retention session two months later in terms of speed (*F*_1,21_  = 1.60, *P* = .2199; *F*_1,20_  = 2.21, *P* = .1529; judgment and production tasks, respectively). In terms of accuracy, there was good retention in the production task (*F*_1,21_  = 1.3, *P* = .1039) but not in the judgment task (*F*_1,21_  = 6.49, *P* = .0188), although the deterioration of performance with time was very small (Fig. 2). There was no significant interaction between age group and retention in terms of accuracy for both tasks and in speed for the judgment task; however, there was a significant interaction in terms of speed in the production task (*F*_2,20_  = 3.85, *P* = .0386), indicating that 8-year-olds showed less retention of speed gains in the production task compared to the older learners (Fig. 2). Thus, while the 12-year-olds and adults robustly retained the speed gains in the production task, the 8-year-olds showed poor performance across the end of the retention interval.

The group average learning curves for each age group were well fitted by a power function model with R^2^ values ranging from 0.82 to 0.96 (p<0.0001) for both performance speed and accuracy, in both tasks. The adults demonstrated the steepest learning curves. The slopes of the regression lines for the 8-year-olds were significantly less steep compared to those of both 12-year-olds [accuracy: (*t*_(16)_  = 4.18, *P*<.001); *t*_16_  = 3.31, *P*<.005); speed: (*t*_16_  = 2.23, .01*<P*<.025; *t*_16_  = 3.1, *P*<.005); judgment and production tasks, respectively] and adults [accuracy: (*t*_(16)_  = 2.395, .01*<P*<.025); speed: (*t*_16_  = 5.18, *P*<.001; *t*_16_  = 16.7, *P*<.001); judgment and production tasks, respectively]. The slopes of the regression lines for the 12-year-olds were less steep compare to those for adults in terms of speed (*t*_16_  = 5.07, *P*<.001); *t*_16_  = 6.8, *P*<.001) but not in terms of accuracy.

**Figure 4:**
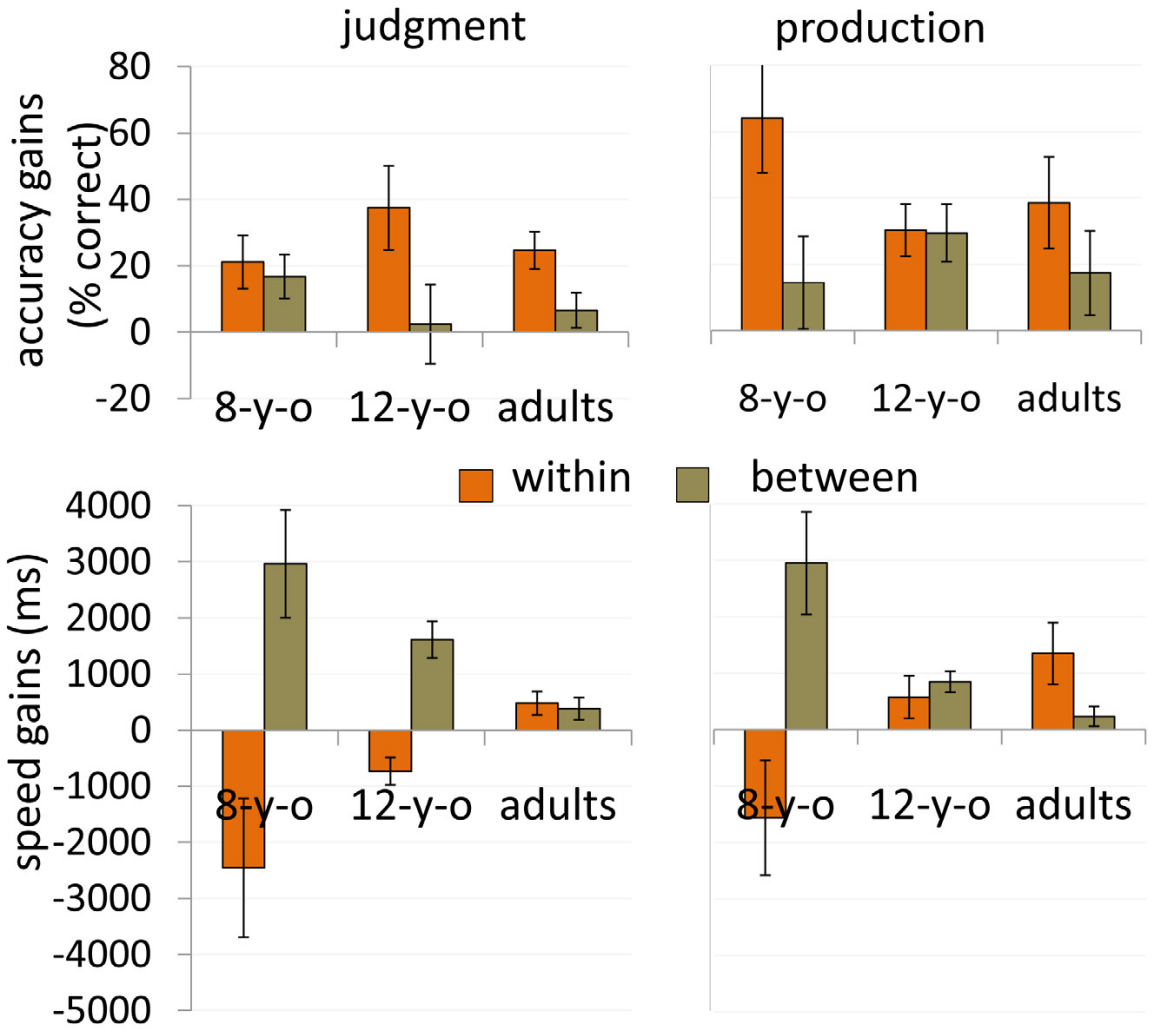
Within and between performance gains. Absolute gains in performance on repeated items attained within-sessions and between-sessions in eight-year-olds (8-y-o), 12-year-olds (12-y-o) and young adults (adults). Judgment (left panels) and speech production (right panels) tasks. Accuracy gains (% correct) - top panels; speed gains (ms) - bottom panels. Error bars  =  standard errors.

In all three age groups, some gains in performance were obtained during the intervals between sessions (delayed performance gains) (Fig. 4). There was a significant age group effect in terms of speed for both the between-sessions gains (*F*_2,21_  = 4.7, *P* = .0206; *F*_2,21_  = 4.88, *P* = .0181, judgment and production tasks, respectively) and within-sessions gains (*F*_2,21_  = 4.24, *P* = .0283; *F*_2,21_  = 4.5, *P* = .0237, judgment and production tasks, respectively), with larger between-sessions gains in 8-year-olds compared to adults and larger within-sessions gains in adults as compared to 8-year-olds. There was no significant age effect for either within- or between-sessions gains in terms of accuracy of performance.

### New items

For new items, all of the 8-year-olds remained at chance level (60% correct and below) in both tasks throughout the training period, i.e., they were unable to generalize the AMR to novel items (Fig. 3). However, the majority of adults (7/8) and 12-year-olds (7/8) attained above 90% correct performance on new items in both the judgment and production tasks (*Table S1*). There was a main effect for session in terms of accuracy in both tasks (*F*_9,178_  = 2.96, *P* = .0026; *F*_9,178_  = 6.9, *P*<.0001) and in terms of speed in the production task (*F*_9,166)_  = 2.87, *P* = .0036) but not in the judgment task (*F*_9,178_  = 1, *P*<.4431). There was also a significant age group effect [accuracy: (*F*_2,21_  = 13.22, *P* = .0002; *F*_2,21_  = 16.08, *P*<.0001); speed: (*F*_2,21_  = 6.08, *P*  = .0052; *F*_2,21_  = 7.06, *P* = .045), judgment and production tasks, respectively]. A significant interaction between age group and session was found for speed in the judgment task (*F*_18,178_  = 1.77, *P* = .0321) with adults showing larger gains compared to the 8-year-olds (*t*_21_  = −4.38, *P* = . 0008) and to the 12-year-olds (*t*_21_  = 3. 4, *P* = .008). When the data were split by age group, there was a main effect for session in terms of accuracy in the production task in 8-year-olds (*F*_9,178_  = 2.01, *P* = .0407), 12-year-olds (*F*_9,178_  = 3.48, *P* = .0006) and adults (*F*_9,178_  = 3.46, *P* = .0006) and in the judgment task in 12-year-olds (*F*_9,178_  = 2.15, *P* = .0276). In terms of performance speed, there was a main effect for session in the judgment task in 8-year-olds (*F*_9,178_  = 2.26, *P* = .0202) and adults (*F*_9,178_  = 1.79, *P* = .0728) and in the production task in 8-year-olds (*F*_9,178_  = 3.78, *P* = .0002).

For the children who practiced 5 sessions more than the adults, a Wilcoxon Signed Rank Test showed that the performance in session 10 was not significantly different from that in session 15 in terms of both speed and accuracy in the two tasks. A Kruskal-Wallis non-parametric analysis of variance showed a significant difference among the three age groups in the final practice session (i.e., session 15 for 8-year-olds and 4/8 of the 12-year-olds and session 10 in adults and 4/8 of the 12-year-olds) in terms of speed and accuracy in both tasks (Fig. 3). Thus, eventually, the children remained inaccurate (by chance) and slower compared to adults in judging and producing the new items (Fig. 3).

In all three age groups tested, average performance in the final practice session did not differ significantly from performance in the retention session two months later [accuracy: (*F*_1,20_  = 0.01, *P* = .9201; *F*_1,20_  = 0.70, *P* = .4127); speed: (*F*_1,20_  = 0.00, *P* = .9933; *F*_1,20_  = 0.00, *P* = .9915), judgment and production tasks, respectively]. Thus, while the 12-year-olds and adults robustly retained the gains in both tasks, the 8-year-olds showed poor performance across both ends of the retention interval (Fig. 3).

### Phonological competence

**Figure 5:**
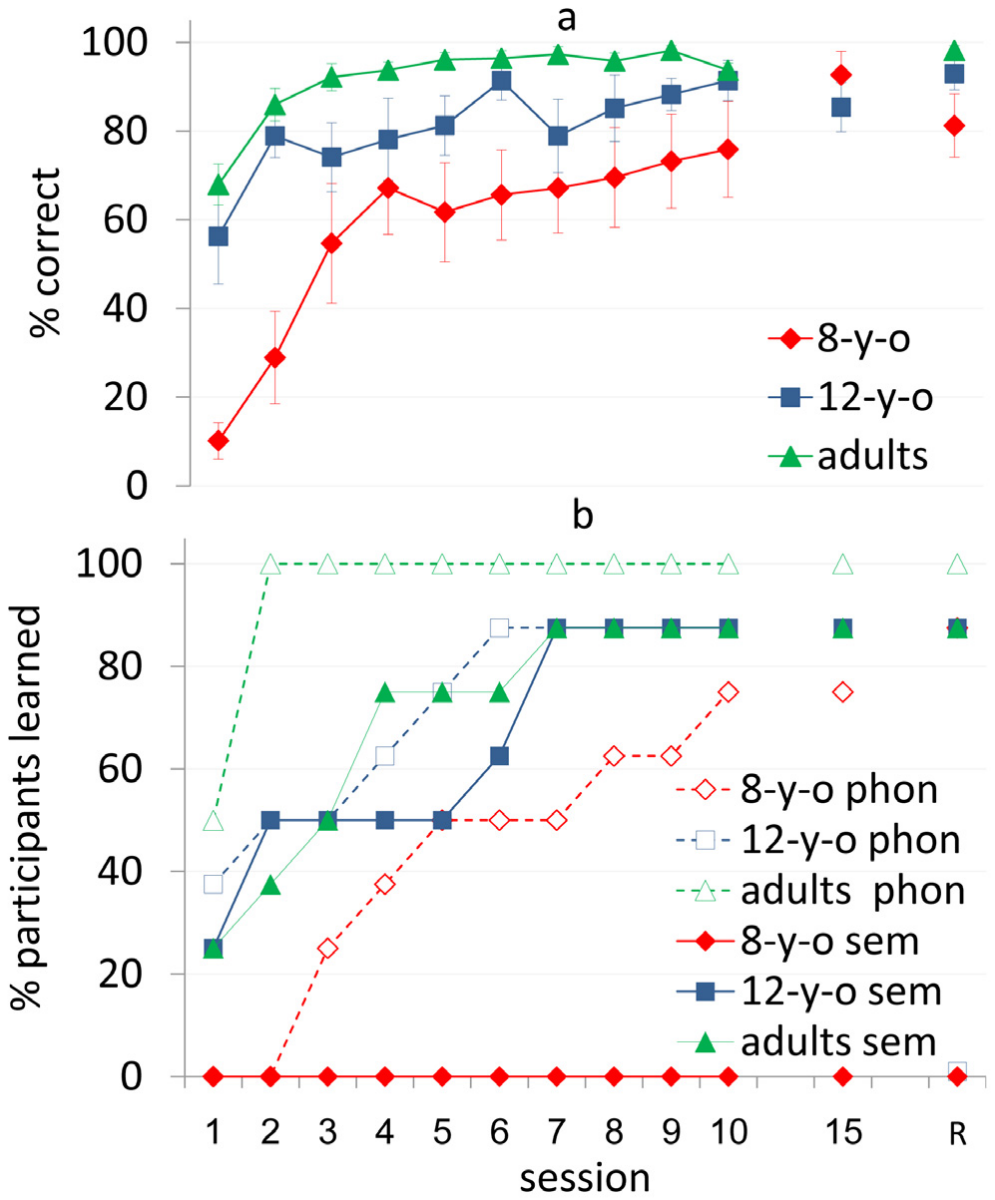
Accuracy of phonological performance (new items) for the 3 age-groups. a - Group average accuracy of phonological performance in 10 consecutive practice sessions, the 15^th^ session, and a retention session (R) in eight-year-olds (8-y-o), 12-year-olds (12-y-o) and young adults (adults). Error bars  =  standard errors. b - Accumulated percentage of participants who acquired the phonological (phon) and semantic (sem) aspects of the AMR.

Correct pronunciation of the target verbs (including the AMR determined suffixes), irrespective of semantic accuracy, was taken as a measure for phonological competence. Target verb pronunciation robustly improved in all three age groups, with all participants acquiring the phonological aspect of the AMR very early on in training (Fig. 5a,b). The analysis showed a main effect for session (*F*_(9,178)_  = 10.21, *P*<.0001) and a significant age group effect (*F*_2,21_  = 11.61, *P* = .0004) with adults outperforming the 8-year-olds (*t*_21_  = −3.92, *P* = .0023) but not the 12-year-olds (*t*_21_  = −1.23, *P* = .5014) and the 12-year-olds outperforming the 8-year-olds (*t*_21_  = −2.65, *P* = .0438). There was also a significant interaction between session and age group (*F*_18,178_  = 1.91, *P*<. 0174), indicating that the adults' gains were larger compared to those of the 8-year-olds, irrespective of the baseline performance (*t*_21_  = 6.47, *P*<.0001; *t*_21_  = 7.32, *P*<.0001).

The group average learning curves for phonological performance were well fitted by a power function model, with R^2^ values ranging from 0.75 to 0.87 (p<.0001) in all three age groups. The phonological performance gains were robustly retained as found on re-testing after a two-month interval in all three age groups (*F*_1,21_  = 2.44, *P* = .1343).

Correct pronunciation of the target verbs, i.e., actual production of the phonological aspect of the AMR, was better and more complete compared to the verbal reports on the nature of the AMR. The participants' explicit reports on the source of their success showed that even when they pronounced the new verbs correctly (>75% phonological correct), their ability to explicitly describe their performance (i.e., describe what they were producing) was at best partial [47]. Early on in training, all individuals reported the addition of the final consonant in the artificial suffixes (/*v*/or/*r*/) while only 3/8 of the adults, 2/8 of the 12-year-olds, and none of the 8-year-olds reported the vowels.

### Semantic aspect

At different time-points between the first and the eighth practice sessions, 7/8 of the adults and 7/8 of the 12-year-olds explicitly reported the semantic distinction (animate, inanimate) as the basis of the AMR. At that time or shortly after, accuracy on new items increased abruptly to more than 90% and 80% correct performance in the judgment and production tasks, respectively. However, none of the 8-year-olds was able to report on the semantic aspect of the AMR or to exceed 65% accuracy in the new items throughout the study (*Table S1*) Most adults and 12-year-olds uncovered the semantic aspect of the AMR early on in training (Fig. 5b).

## Discussion

Altogether, the current results clearly show that maturation had a positive effect on the acquisition and retention of an artificial language skill when an equivalent language experience was afforded to 8-year-olds, 12-year-olds and young adults. Young adults outperformed both groups of children and 12-year-olds outperformed the 8-year-olds. The adults' advantage over the 8-year-olds was clear even after the latter were given five additional practice sessions. The age-related advantage was expressed not only in the initial level of performance but also in the rate of improvement across the training sessions and in the effectiveness of retention at two months post-training. Furthermore, the age-related advantage was expressed in all performance parameters tested, including task aspects that were clearly implicit. This advantage was reflected not only in the ability to judge the repeated items, but also in the ability to produce them and in the ability to correctly pronounce the artificial phonological pattern when generalizing the AMR to novel items - aspects of linguistic competence that were reported to reflect a childhood advantage [23], [27]–[28]. Although training was conducted by means of exposure to and use of the AMR and there was no explicit instruction of the AMR at any time during the training, only the older learners were able to uncover the semantic aspect of the AMR through linguistic experience.

Throughout the practice sessions, the age-related advantage was reflected not only in higher accuracy of performance, but also in more fluent performance (i.e., shorter response times) in both the judgment and the production task. The finding of an advantage in fluency (speed) of production rather than an advantage in judgment and performance accuracy per se in a linguistic task is in line with recent findings in linguistic [52] and non-linguistic [53] skill learning that children are slower compared to adults.

The current findings suggest that learning to apply an AMR to repeated (specific) items as well as to fluently produce the phonological patterns required by the AMR when applied to novel items, demonstrate key characteristics of procedural learning [54]. Competence in these aspects of the task was acquired implicitly, with power-law like group average gains in speed and accuracy [13]–[14], [48]–[49], [55], no speed accuracy trade-off [56]–[57] and robust long-term retention of the gains [12]–[14], [55]–[56] (Fig. 2). The current results also show that some of the gains in both the judgment and production of repeated items evolved between sessions, i.e., in the post-training intervals, rather than concurrently with practice (Fig. 4). These delayed (“off-line”) gains, occurred in both the 8- and the 12-year-olds as well as in the course of learning the AMR in adults. Delayed gains in task performance were proposed to reflect procedural memory consolidation processes in a number of perceptual and motor skill learning paradigms [13]–[14], [48]–[49], [56], [58]. Thus, the results of the current study provide a behavioral indication for a consolidation phase in linguistic learning in pre-adolescent children as well as in young adults. This phase, we propose, may correspond to the procedural memory consolidation phase as recently reported, for children and young adults in a motor sequence learning task [50]. Thus, our results are not consistent with a simple version of the proposal that procedural memory, per se, is less effective in adults compared to children [5]–[6].

The current results suggest that the discovery of the semantic (animate-inanimate) distinction and its requisite role in the AMR was crucial for accurate generalization to new items. Furthermore, the results indicate that the acquisition of the semantic aspect of the AMR was in the form of an explicit discovery, i.e., the establishment of declarative knowledge [10]–[11]. The explicit verbal reports on the role of the semantic distinction in the AMR co-occurred, within a single session, with abrupt increases in the accuracy scores and sometimes with abrupt, transient decreases in speed, in the performance of new items [54]. This knowledge was well retained in memory. The accuracy of performance in the application of the AMR to new items continued to increase in subsequent sessions with eventually, almost perfect performance. However, unlike the majority of adults and 12-year-olds, the 8-year-olds failed to establish explicit knowledge of the semantic aspect of the AMR and subsequently to generalize it accurately to novel items. This is in line with the notion of maturation, across childhood and early adolescence, of the declarative memory system [17]–[19]. It has been suggested that children are less able than adults to use lexical–semantic cues during grammatical processing (Shallow Processing Hypothesis) [52].

The older participants may have also benefited from better working memory resources, more mature problem solving strategies [59], as well as from their previous, more extensive linguistic experience, including with morphological rules. Although the stimuli were artificial, the phonological features were compatible with the participants' native language, and the AMR was akin to typical morphological rules in Hebrew. Older participants may therefore have been more familiar with the notion that semantic distinctions can be conveyed through phonological patterns.

It has been proposed that a childhood advantage in language rule acquisition, if present, is related to the dominance of implicit (procedural) learning mechanisms [5]–[6]. According to this view, there is an inherent advantage contingent on the immaturity of the explicit memory system in children. Our results however, may be taken as support for the notion that effective explicit learning abilities may in fact be helpful in learning (artificial) language rules and therefore, children being largely limited to implicit learning are at a disadvantage rather than an advantage [17]–[19], [59]. Older children and adults who presumably possess a more mature declarative memory system were superior to the younger children in acquiring the implicit (procedural) aspects of the language task as well as in discovering the underlying semantic distinction, both implicitly (as expressed in actual performance) and explicitly (overt report).

The current results, however, suggest an age-related improvement (between ages 8 and young adulthood) not only in the explicit discovery of the semantic aspect of the AMR, but also in the procedural learning of language aspects, including phonology. Several previous studies have already suggested that procedural memory for linguistic skills may not be fully developed in childhood; these include studies on language attrition [60] second language acquisition [28]-[34], [44], [46] and children with cochlear implants [61], where comparable learning conditions were available to older and younger learners. Our results, therefore, are in line with recent evidence suggesting that the procedural memory system may undergo maturation across childhood and well into adulthood in humans [17]–[19], [50], [59] and animals [62]. Recent studies using the AMR and training paradigm as in the current study, showed that some, but not all, 8-year-olds were able to acquire the semantic aspect of the AMR and to generalize it to new items when the semantic aspect was made more salient in the training conditions. Nevertheless, even under these conditions, the younger children were outperformed by older participants, with the adults gaining superior fluency and accuracy (Ferman & Karni, “The effect of age and type of training material on the ability to learn an AMR”, Proceedings of the 27^th^ World Congress of ILAP, 2007, Copenhagen, E-address: www.ialp.info; Ferman & Karni, submitted).

Our results do not support the notion that while language abilities in children evolve slowly, children outperform adults in the long run [3], [22], [63]. We followed the AMR learning process intensively for over 3 months (5–6 weeks of multi-session training and a retention test given 9 weeks after the termination of training), and adults were superior in both attainments and learning rates. However, given that the current language task was only applicable in the laboratory, our results cannot be construed as incompatible with the notion that given continuous exposure, children may excel in the task in subsequent months and years.

Our findings of clear age-related advantages between age 8 and young adulthood in learning a new morphological skill do not support a simple notion of a restricted developmental time window or a ‘critical period’ of heightened plasticity in linguistic skill acquisition. The finding that adults have effective language skill learning and express effective procedural memory for a language task, albeit in a laboratory setting, is a good indication that the basic mechanisms of skill acquisition (i.e., implicit learning) are not lost to young adults in the domain of language competence; our data suggest that the potential for language skill acquisition may even be superior to that available before puberty. This notion, however, is at odds with the substantial evidence indicating a childhood advantage in language skill learning [1]–[3], [6], [20], [22], [24]. Our proposal states that there are two separate issues: the availability of effective skill learning mechanisms, in the domain of language competence, in adulthood and the effects of experiential factors that may block their full expression.

It was recently proposed [50] that while there is no childhood advantage in the acquisition, consolidation, and retention of motor skills, the consolidation of procedural memory for such skills may be less prone to interference by subsequent experience before puberty. In adults, the establishment of new skills may, under some conditions, be interfered with even by subsequent experience with a previously acquired well-established skill. Thus, it may be the case that in situations in which interference is minimized or absent, the adults' performance and learning advantages can become apparent, whereas in situations in which interference is ubiquitous, the adults' potential for learning cannot be fully expressed. One would hypothesize that an instance of the former type of conditions would be ‘immersion’ in a new language, in which case an adult advantage would be expected. However, whenever the exposure to a new language is closely followed by exposure to a previously well-established language, one would predict that adults would be disadvantaged relative to children. Rather than a simple notion of an irreversible loss of plasticity in adults, this proposal may provide an alternative explanation for the apparently conflicting results regarding the ability of children and adults to acquire linguistic skills. Thus, the adult ‘disadvantage’, found in some conditions, may reflect an inability to establish long-term memory given the specific structure of the learning experience. Other mechanisms, such as proactive interference, whereby previously established knowledge may compete and even interfere with subsequent learning, may also be at work in late learners and in some instances result in an early learning advantage [50].

This proposal may establish a correspondence between linguistic and non-linguistic skill acquisition; in the latter case, there is ample evidence for highly effective procedural memory in adults, including experience-dependent neuronal changes in low-level processing areas [13]–[14], [48]–[49]. Effective skill acquisition in adulthood was also shown in animal studies [62]. There is evidence suggesting, therefore, that rather than an irreversible loss of plasticity in adults, adult skill learning may be more strictly controlled than (but as effective as) skill acquisition before puberty [17]–[19], [50].

In this study we used a laboratory artificial language paradigm in laboratory settings. The advantages of doing so reside in the ability to ensure that the material to be learned is equally new for all learners and that the exposure to input, instructions, and tasks are identical. Furthermore, the constrained design of an artificial language makes it possible to isolate specific language features from the complex interactions found in natural language, and to manipulate them in order to study them separately [64]–[65]. Laboratory settings afford fine-grained data collection, in real time, that cannot be achieved in real life language learning situations [66]–[67]. On the other hand, one may argue that, the simplified language and laboratory environment in artificial language paradigms cannot and do not express the complexity neither of natural language nor of real-life learning conditions. This needs to be taken into account in the translation of any laboratory intervention to ‘real-life’ learning.

Rather than use an artificial language, we used an artificial rule that can be considered a partial artificial language paradigm [66]. One of the benefits of such a paradigm is that the trained task is related to previously established language knowledge (e.g., identity of lexical items); it therefore resembles non-linguistic laboratory skill learning paradigms in which the novel skill builds upon existing skills (e.g., finger opposition [49]; voice in noise discrimination, [48]). The participants made phonological, morphological, and semantic errors that clearly indicated a reliance on their native language linguistic experiences [54]. Thus, the current experimental paradigm can be considered as a laboratory paradigm for morphological acquisition in late stages of first language development [68], or second language acquisition [69].

Our laboratory approach was based on similar experiments in which the acquisition of perceptual or motor skills was studied (in humans or animals) [12–14, 48–50 55–56]. The claim is not that laboratory settings mimic real-life situations; in fact, the conditions are often chosen so as to represent ‘novel’ experiences in the tested domain. The underlying assumption is, however, that memory mechanisms engaged in the laboratory are the same mechanisms available in natural settings – that is, the same basic learning mechanisms should be at work. The drawing of theoretical and practical implications from the present laboratory study, however, should be cautious; broader, ecologically valid data, are needed in order to understand when and under what conditions this potential for language learning can be effectively expressed at different stages of development.

Altogether the current findings may be interpreted to reflect an age-related maturation, between childhood and adulthood, of both the declarative and the procedural memory systems in the context of acquiring a new linguistic skill. Under our laboratory conditions, maturation or accumulated experience, or both, between childhood and adulthood had a positive effect on the ability to learn each and every aspect of the language task.

We propose that the current data support the availability of effective language skill learning mechanisms in adults. Our laboratory settings, however, cannot address the possibility that adults' potential to learn new language skills may not be fully or partially expressed in many natural settings, as well as in specific laboratory conditions, because of factors other than the loss of skill learning abilities per se [50]. The implication, which is empirically testable, is that in some conditions, adults are expected to manifest advantages in language skill acquisition, while in other conditions, they may do worse than children. The apparent childhood advantages, reported in many studies, may therefore reflect the effect of structural aspects of everyday language learning experiences that afford less than optimal conditions for adults to fully express their competence in skill (implicit) acquisition and procedural memory.

## Supporting Information

Table S1 Individual accuracy performance gains in the new items. Percent correct performance of each individual participant (I.P.) for the new items in the judgment (jud) and production (pro) tasks in the 10 initial sessions, at the 15th session and in the retention session (R), in 8-year-olds (S1a), 12-year-olds (S1b) and adults (S1c).
(0.14 MB DOC)
