# Supplementary material for: No Childhood Advantage in the Acquisition of Skill in Using an Artificial Language Rule
Source: PLoS One. 2010 Oct 27;5(10):e13648. doi: 10.1371/journal.pone.0013648 (PMC2965096; doi:10.1371/journal.pone.0013648)
Supplement: Table S1 — Individual accuracy performance gains in the new items. Percent correct performance of each individual participant (I.P.) for the new items in the judgment (jud) and production (pro) tasks in the 10 initial sessions, at the 15th session and in the retention session (R), in 8-year-olds (S1a), 12-year-olds (S1b) and adults (S1c). (0.14 MB DOC) [file pone.0013648.s001.doc]

**Table S1 - Individual accuracy performance gains in the new items.**

**S1a- 8-year-olds**

| **session** | | **1** | **2** | **3** | **4** | **5** | **6** | **7** | **8** | **9** | **10** | **15** | **R** |
| --- | --- | --- | --- | --- | --- | --- | --- | --- | --- | --- | --- | --- | --- |
| **I.P.** | **task** |  |  |  |  |  |  |  |  |  |  |  |  |
| E.C. | jud | 37.5 | 56.2 | 50 | 50 | 43.7 | 62.5 | 62.5 | 37.5 | 50 | 37.5 | 37.5 | 43.7 |
|  | pro | 0 | 12.5 | 18.7 | 43.7 | 31.2 | 25 | 6.2 | 25 | 12.5 | 25 | 68.7 | 50 |
| B.K. | jud | 50 | 56.2 | 37.5 | 31.2 | 25 | 56.2 | 87.5 | 93.7 | 87.5 | 93.7 | __ | 83.7 |
|  | pro | 43.7 | 50 | 37.5 | 37.5 | 37.5 | 81.2 | 75 | 68.3 | 56.2 | 56.2 | __ | 50 |
| D.B. | jud | 50 | 50 | 68.8 | 50 | 43.7 | 56.2 | 43.7 | 43.7 | 43.7 | 68.7 | 62.5 | 43.7 |
|  | pro | 6.2 | 25 | 18.7 | 50 | 50 | 25 | 37.5 | 56.2 | 31.2 | 37.5 | 31.2 | 31.2 |
| R.D. | jud | 28.1 | 37.5 | 56.2 | 37.5 | 50 | 50 | 56.2 | 37.5 | 43.7 | 68.7 | 62.5 | 43.7 |
|  | pro | 6.2 | 18.7 | 6.2 | 37.5 | 43.7 | 25 | 37.5 | 37.5 | 37.5 | 37.5 | 56.2 | 50 |
| E.S. | jud | 62.5 | 68.7 | 43.7 | 37.5 | 56.2 | 43.7 | 43.7 | 56.2 | 50 | 56.2 | 31.2 | 56.2 |
|  | pro | 0 | 0 | 43.7 | 25 | 18.7 | 25 | 18.7 | 31.2 | 25 | 43.7 | 56.2 | 50 |
| E.T. | jud | 50 | 68.7 | 62.5 | 31.2 | 62.5 | 68.7 | 56.2 | 37.5 | 68.7 | 68.7 | 43.7 | 56.2 |
|  | pro | 6.2 | 31.2 | 50 | 43.7 | 31.2 | 31.2 | 25 | 37.5 | 43.7 | 56.2 | 50 | 62.5 |
| L.B. | jud | 43.7 | 50 | 50 | 56.2 | 43.7 | 50 | 43.7 | 56.2 | 43.7 | 56.2 | 56.2 | 62.5 |
|  | pro | 0 | 6.2 | 6.2 | 12.5 | 12.5 | 18.7 | 12.5 | 18.7 | 18.7 | 37.5 | 37.5 | 43.7 |
| A.N. | jud | 50 | 68.7 | 62.5 | 50 | 50 | 25 | 50 | 56.1 | 43.7 | 68.7 | 43.7 | 37.5 |
|  | pro | 0 | 12.5 | 12.5 | 12.5 | 0 | 0 | 0 | 0 | 6.2 | 6.2 | 25 | 6.2 |

**S1b- 12-year-olds**

| **session** | | **1** | **2** | **3** | **4** | **5** | **6** | **7** | **8** | **9** | **10** | **15** | **R** |
| --- | --- | --- | --- | --- | --- | --- | --- | --- | --- | --- | --- | --- | --- |
| **I.P.** | **task** |  |  |  |  |  |  |  |  |  |  |  |  |
| B.S. | jud | 43.7 | 43.7 | 37.5 | 68.7 | 56.2 | 50 | 68.7 | 62.5 | 75 | 62.5 | 43.7 | 43.7 |
|  | pro | 25 | 25 | 25 | 25 | 43.7 | 25 | 12.5 | 25 | 18.7 | 31.2 | 37.5 | 56.2 |
| E.D. | jud | 56.2 | 50 | 43.7 | 31.2 | 50 | 37.5 | **87.5*** | 100 | 87.5 | 75 | 75 | 68.7 |
|  | pro | 31.2 | 50 | 50 | 43.5 | 37.5 | 37.5 | 62.5 | 100 | 93.7 | 81.2 | 87.5 | 81.2 |
| L.N. | jud | **93.7*** | 81.2 | 93.7 | 75 | 87.5 | 87.5 | 87.5 | 100 | 100 | 87.5 | __ | 87.5 |
|  | pro | 81.2 | 93.7 | 87.5 | 100 | 93.7 | 87.5 | 93.7 | 93.7 | 93.7 | 100 | __ | 87.5 |
| L.A. | jud | 25 | **50*** | 87.5 | 90.6 | 93.7 | 87.5 | 87.5 | 93.7 | 62.5 | 100 | 100 | 100 |
|  | pro | 0 | 43.7 | 62.5 | 62.5 | 50 | 68.7 | 75 | 75 | 50 | 81.2 | 81.2 | 75 |
| M.C. | jud | 25 | 50 | 56.2 | 25 | 43.7 | 50 | **56.2*** | 62.5 | 93.7 | 93.7 | 93.7 | 81.2 |
|  | pro | 12.5 | 25 | 37.5 | 37.5 | 25 | 56.2 | 43.7 | 37.5 | 87.5 | 100 | 100 | 100 |
| M.K. | jud | 68.7 | 43.7 | 62.5 | 43.7 | 25 | **100*** | 100 | 100 | 100 | 87.5 | __ | 100 |
|  | pro | 12.5 | 31.2 | 31.2 | 31.2 | 31.2 | 43.7 | 56.2 | 75 | 75 | 87.5 | __ | 100 |
| N.V. | jud | **100*** | 100 | 100 | 100 | 100 | 100 | 100 | 93.7 | 100 | 100 | __ | 100 |
|  | pro | 93.7 | 93.7 | 100 | 100 | 100 | 100 | 93.75 | 100 | 100 | 100 | __ | 100 |
| L.B. | jud | 31.2 | **68.7*** | 100 | 100 | 87.5 | 93.7 | 100 | 93.7 | 100 | 93.7 | __ | 87.5 |
|  | pro | 25 | 62.5 | 62.5 | 100 | 81.2 | 87.5 | 100 | 100 | 87.5 | 100 | __ | 100 |

**S1c – Adults**

| **session** | | **1** | **2** | **3** | **4** | **5** | **6** | **7** | **8** | **9** | **10** | **15** | **R** |
| --- | --- | --- | --- | --- | --- | --- | --- | --- | --- | --- | --- | --- | --- |
| **I.P.** | **task** |  |  |  |  |  |  |  |  |  |  |  |  |
| G.L. | jud | 43.7 | 37.5 | ***100** | 100 | 100 | 100 | 100 | 93.7 | 100 | 100 | __ | 100 |
|  | pro | 31.2 | 50 | ***100** | 100 | 87.5 | 93.7 | 100 | 93.7 | 93.7 | 93.7 | __ | 100 |
| H.M. | jud | 68.7 | 56.2 | ***75** | 93.7 | 100 | 93.7 | 100 | 100 | 87.5 | 93.7 | __ | 100 |
|  | pro | 25 | 50 | ***68.7** | 87.5 | 81.2 | 93.7 | 93.7 | 75 | 93.7 | 87.5 | __ | 100 |
| N.N. | jud | ***93.7** | 93.7 | 100 | 93.7 | 100 | 100 | 100 | 100 | 93.7 | 93.7 | __ | 100 |
|  | pro | ***87.5** | 100 | 87.5 | 87.5 | 93.7 | 100 | 93.7 | 93.7 | 93.7 | 87.5 | __ | 100 |
| R.B. | jud | 50 | 50 | 56.2 | ***100** | 100 | 100 | 93.7 | 93.7 | 93.7 | __ | __ | 100 |
|  | pro | 31.2 | 25 | 75 | ***93.7** | 100 | 93.7 | 87.5 | 93.7 | 100 | __ | __ | 93.8 |
| T.G. | jud | ***75** | 93.7 | 100 | 100 | 100 | 100 | 100 | 100 | 93.7 | __ | __ | 100 |
|  | pro | ***43.7** | 62.5 | 81.2 | 100 | 93.7 | 87.5 | 93.7 | 100 | 100 | __ | __ | 100 |
| Y.L. | jud | 68.7 | ***100** | 100 | 100 | 93.7 | 93.7 | 100 | 93.7 | 100 | 100 | __ | 100 |
|  | pro | 56.2 | ***81.2** | 93.7 | 100 | 87.5 | 87.5 | 93.75 | 100 | 100 | 100 | __ | 100 |
| Z.A. | jud | 43.7 | 37.5 | 25 | 43.7 | 68.7 | 56.2 | 43.7 | ***100** | 100 | 93.7 | __ | 87.5 |
|  | pro | 31.2 | 43.7 | 31.2 | 56.2 | 68.7 | 31.2 | 31.2 | ***87.5** | 93.7 | 87.5 | __ | 81.2 |
| E.S. | jud | 43.7 | 59.3 | 62.5 | 43.7 | 43.7 | 62. | 56.2 | 56.2 | 43.7 | 56.2 | __ | 56.2 |
|  | pro | 43.7 | 43.7 | 43.7 | 50 | 62.5 | 43.7 | 62.5 | 43.7 | 43.7 | 25 | __ | 37.5 |

* The session wherein the participant explicitly reported the semantic distinction.
